# Impact of 24 h shifts on urinary catecholamine in emergency physicians: a cross-over randomized trial

**DOI:** 10.1038/s41598-024-58070-2

**Published:** 2024-03-27

**Authors:** Frédéric Dutheil, Alicia Fournier, Christophe Perrier, Damien Richard, Marion Trousselard, George Mnatzaganian, Julien S. Baker, Reza Bagheri, Martial Mermillod, Maelys Clinchamps, Jeannot Schmidt, Jean-Baptiste Bouillon-Minois

**Affiliations:** 1 CNRS, LaPSCo, Physiological and Psychosocial Stress, CHU Clermont-Ferrand, Occupational and Environmental Medicine, Wittyfit, Université Clermont Auvergne, Clermont-Ferrand, France; 2 Psy-DREPI Laboratory UR 7458, University of Bourgogne, Dijon, France; 3 Emergency Department, CHU Clermont-Ferrand, Clermont-Ferrand, France; 4 Unité INSERM 1107 Neuro-Dol, CHU Clermont-Ferrand, Université Clermont-Auvergne, 63000 Clermont-Ferrand, France; 5 Neurophysiology of Stress, Neuroscience and Operational Constraint Department, French Armed Forces Biomedical Research Institute (IRBA), 91223 Brétigny-sur-Orge, France; 6 Rural Department of Community Health, La Trobe Rural Health School, College of Science, Health and Engineering, La Trobe University, Melbourne, VIC Australia; 7 Department of Sport, Physical Education and Health, Center for Health and Exercise Science Research, Hong Kong Baptist University, Kowloon Tong, Hong Kong; 8 Department of Exercise Physiology, University of Isfahan, Isfahan, 81746-73441 Iran; 9 CNRS, LPNC, Grenoble, France Institut Universitaire de France, Univ. Grenoble Alpes, Univ. Savoie Mont Blanc, Paris, France; 10 Emergency Department, CNRS, LaPSCo, Physiological and Psychosocial Stress, CHU Clermont-Ferrand, Université Clermont Auvergne, 63000 Clermont-Ferrand, France

**Keywords:** Health-care workers, Stress, Epinephrine, Dopamine, Sleep, Shift work, Biomarkers, Health occupations, Medical research, Neurology, Risk factors

## Abstract

24-h shift (24 hS) exposed emergency physicians to a higher stress level than 14-h night shift (14 hS), with an impact spreading on several days. Catecholamines are supposed to be chronic stress biomarker. However, no study has used catecholamines to assess short-term residual stress or measured them over multiple shifts. A shift-randomized trial was conducted to study urinary catecholamines levels of 17 emergency physicians during a control day (clerical work on return from leave) and two working day (14 hS and 24 hS). The Wilcoxon matched-pairs test was utilized to compare the mean catecholamine levels. Additionally, a multivariable generalized estimating equations model was employed to further analyze the independent relationships between key factors such as shifts (compared to control day), perceived stress, and age with catecholamine levels. Dopamine levels were lower during 24 hS than 14 hS and the control day. Norepinephrine levels increased two-fold during both night shifts. Epinephrine levels were higher during the day period of both shifts than on the control day. Despite having a rest day, the dopamine levels did not return to their normal values by the end of the third day after the 24 hS. The generalized estimating equations model confirmed relationships of catecholamines with workload and fatigue. To conclude, urinary catecholamine biomarkers are a convenient and non-invasive strong measure of stress during night shifts, both acutely and over time. Dopamine levels are the strongest biomarker with a prolonged alteration of its circadian rhythm. Due to the relation between increased catecholamine levels and both adverse psychological effects and cardiovascular disease, we suggest that emergency physicians restrict their exposure to 24 hS to mitigate these risks.

## Introduction

Emergency medicine involves a complex interplay between stress, sleep deprivation, and fatigue^1^. Complexities are exacerbated by repeated changing length of shifts^2^. Emergency physicians (EPs) are exposed to work-related exhaustion including detachment from work, feelings of diminished competence, delayed decision-making and medical errors^1,2^. These factors collectively contribute to the early departure of EPs^3^. Moreover, prolonged stress exposes EPs to a higher risk of multiple diseases^4^. Long working hours has been showed to increase blood biomarkers such as cholesterol, triglycerides, glucose, gamma-glutamyl transferase, alanine transaminase, white blood cells, creatinine, ghrelin and platelets, and decrease cortisol, leptin and haemoglobins^5–7^.Catecholamines are linked with the exact same diseases^8,9^. It is mainly involved in the acute response to stress, following the activation of the sympathetic nervous system and hypothalamic–pituitary–adrenal axis^10^. However, no studies have compared the impact of durations of shift work on catecholamine levels, nor their evolution on subsequent days following shifts. Norepinephrine and epinephrine increases with physical demands and are used as biomarkers of stress^11^. Specifically, higher norepinephrine and epinephrine levels have been reported in physicians performing a 24-h shift (24 hS) when levels were compared with a control day^12^. Dopamine is the first, rate-limiting step in the biosynthesis of catecholamine^13^ that can be inactivated under the action of specific enzymes, yielding to the production of vanillylmandelic acid (VMA) and homovanillic acid (HVA)^13^. Moreover, mental and physical stress can be dissociated through catecholamine levels^14^. Conveniently, catecholamine levels can be measured non-intrusively and painless using urinary samples^15^. In our previous publication, we reported that senior EPs experienced fatigue and a higher accumulation of stress during a 24 hS compared to a 14-h night shift (14 hS), and these effects persisted for several days^16^. From these observations, we hypothesized that (1) catecholamine levels could serve as a pertinent biomarker for stress and fatigue, with levels exhibiting sufficient sensitivity to differentiate between two durations of night shifts, (2) catecholamines have in a prolonged response after 24 hS compared to 14 hS, (3) dopamine, as a precursor of catecholamine, would decrease with production of norepinephrine and epinephrine, and (4) the complete catecholamine pathway would be associated with stressful events surrounding the shifts. To date, no study has investigated the complete catecholamine pathway in EPs. Therefore, the objective of this study was to evaluate the influence of 14 hS and 24 hS on the urinary catecholamine levels of senior EPs participating in the JOBSTRESS trial (Biomarkers of Job Stress: a Randomized Trial among Senior Emergency Physicians). Secondary objectives were to study its association with fatigue, sleep deprivation, stress, workload, and other variables. We also assessed urinary catecholamine levels 3 days after the completion of each shift to identify any enduring effects that a previous shift may have on the catecholamine pathways.

## Methods

### Participants

We recruited EPs from the University Hospital of Clermont-Ferrand (CHU), France. A comprehensive explanation of the study was provided to each participant. Participants who met any of the following criteria were excluded: endocrine disease, pregnancy, recent life stress event unrelated to the profession, any ongoing illness, use of medications for inflammatory conditions, or with a chronotropic effect within the past 6 months.

### Study design

**Figure 1:**
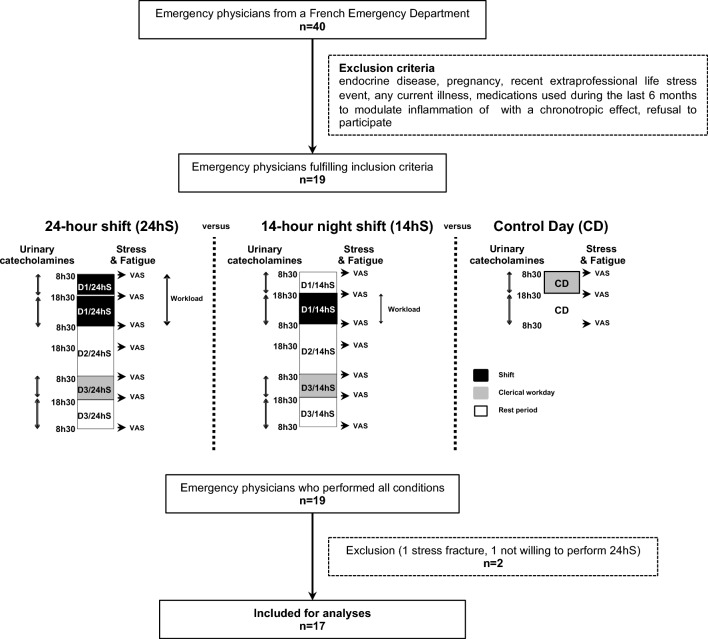
Flowchart including Study Design Experimental protocol: 3 days of follow-up after each type of shift compared with a control day. Catecholamines measured were dopamine, norepinephrine, epinephrine, homo-vanillic acid and vanillin-mandelic acid. *VAS* Visual analog scale.

We compared two types of shifts in day 1 (D1): a 24 hS lasting from 8:30 to 8:30 am the following morning and a 14 hS from 6:30 pm to 8:30 am; the order of shifts was randomized. All shifts were performed in the same emergency department (ED) that accept daily 160 patients. EPs did not work the day before both shifts. Prior to a 14 hS, EPs were at home from 8:30 am to 6:30 pm (Fig. 1). To maintain their usual routine, no instructions were given to participants before commencing the shift. Each shift was followed on day 2 (D2) by a rest day, and then on day 3 (D3) by a day of clerical work. During this day, participants had no contact with patients and were involved in administrative tasks—coding medical procedures, interpreting X-rays and blood sample, preparing lessons. This clerical workday was added to participants’ schedule to avoid bias between comparisons of shifts. Finally, each shift was compared with another control day standardized to be a clerical workday (8:30 am–6:30 pm) after returning from at least 8 days of holiday. On the designated day, participants were instructed to refrain alcohol and caffeinated beverages, and to avoid strong physical activity.

### Assessment of catecholamine

Independent of the shift, EPs were asked to collect their urine in distinct bottles during two separate time periods: from 8:30 am to 6:30 pm and from 6:30 pm to 8:30 am. This was completed during D1 (shifts), D3 (clerical day) and the control day (Fig. 1). We measured the urine volume, and a two mL urine sample was extracted and preserved at a temperature of -80 °C for future analysis. Catecholamine levels were measured by the same researcher (blinded to the type of shift) using high performance liquid chromatography with electrochemical detection (Chromsystems®, Munich, Germany). Sensitivity, intra- and inter-assay coefficients of variation were respectively, 93.8 nmol/l, 2.3%, 5.2% for dopamine, 90.6 nmol/l, 2.9%, 6.9% for norepinephrine, 42.2 nmol/l, 4.3%, 8.5% for epinephrine, 1.9 µmol/l, 2.4%, 11.3% for HVA, and 6.0 µmol/l, 2.9%, 14.2% for VMA.

### Other assessments

Perceived stress was evaluated using a visual analogue scale (VAS). This scale can measure the perceived stress level experienced by individuals in various domains such as work, home, and daily life. It consists of a horizontal line of 100 mm on the paper without specific markings, where the left end represents a very low stress level (0) and the right end a very high stress level (100). We used the same scale to assess mental and physical fatigue. These measurements occurred at 8:30 am and at 6:30 pm on each condition of the protocol. We previously, published results from mental, physical fatigue and perceived stress^17^. An estimation of workload during shift was made using the total number of consults, beds available on the hospital, and life-and-death emergencies. When we compared 24 hS and 14 hS, we limited analyses to workloads between the shared times (6:30 pm to 8:30 am). A questionnaire was used to assess sleep duration (bedtime—wake time), including naps, on the control day, the day prior to the shifts and during the 3-day follow-up of each shift (Fig. 1). Lastly, we assessed sociodemographic (age, gender, and body mass index).

### Statistics

Catecholamine levels were the primary outcome variable. We calculated our sample size on norepinephrine levels from a preliminary study of our team that showed that a difference in catecholamine levels of approximately 20 ± 10% was required to differentiate between a 24 hS and a regular day. We assumed that a difference in catecholamine levels of approximately 20 ± 10% would be required to differentiate between 24 and 14 hS. Based on this difference, we determined a sample size of 10 participants yield a statistical power of 80% with an alpha risk at 5%. However, we recruited all EPs to best represent the whole team of ED. The statistical analyses were conducted using SPSS Advanced Statistics software, version 20 (SPSS Inc., Chicago, IL). Shapiro–Wilk test was used to assess the Gaussian distribution for each parameter. Unless otherwise stated, values are reported in means and standard deviations. Wilcoxon matched pairs (signed rank) tests was used to compared mean catecholamine levels in the three conditions (24 hS, 14 hS and control day). To investigate the independent relationships between fatigue, stress, and sleep deprivation with catecholamine levels, a multivariable generalized estimating equations model was employed. This model controlled for factors such as age, gender, and body mass index (BMI) while considering the variations in correlation between repeated measures. A non-parametric Spearman test was utilized to establish the correlation matrix between the measured parameters. Significance was set at the *p* < 0.05 level. We used Latin squares to randomize the pattern of shifts and the control day. An investigator was present at the beginning and at the end of every shift to increase data completion and urine collection. Thus, we did not have any relevant missing data.

### Ethics

The protocol was approved by the Ethics Committee South East I on 11/01/2010, of the University Hospital of Saint-Etienne, France (ClinicalTrials.gov Identifier NCT01874704 first submitted on 11/06/2013). Each participant provided written an informed consent after presentation of the study by an investigator. The research was performed in accordance with the Declaration of Helsinki.

## Results

### Characteristic of the population

A total of 19 participants were recruited for the study (seven men). Complete data were obtained from 17 physicians. Two were excluded—one suffered from a stress fracture, the other was not willing to perform 24 hS. EPs were 39.1 ± 6.9 years old, with a mean BMI of 22.8 ± 3.1 kg m^2^, and an average seniority of 6.3 ± 4.8 years. Participants were mainly married (65%). Five (29%) smoked. All drank coffee: 5.2 ± 2.4 cups in 24 hS, 3.5 ± 2.0 in 14 hS and 4.8 ± 3.1 on the control day. Thirteen (76%) engaged regularly in physical activity (4.3 ± 2.4 h per week). None drank more than two alcoholic beverages per day. Catecholamine levels were not linked to putative confounding factors such as sociodemographic, coffee, smoking, alcohol, nor physical activity (Fig. 1).

### Catecholamine

**Figure 2:**
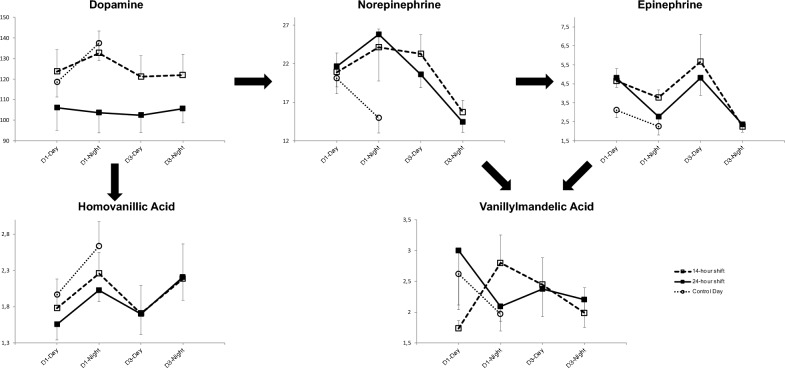
Evolution of urinary catecholamine during the shifts (Day 1) and on the workday (D3) and during the control clerical day. Results are expressed as mean ± standard error. X axis represents the different periods of measurement. Y axis represents the value of catecholamines, expressed in nmol/mol of creatinine. Tick arrows represents the physiology of catecholamines.

More detailed comparisons of night and day catecholamine levels are presented in Fig. 2. Our data have a normal distribution, so were not log transformed. Although our sample seems sample, we have ten measurements time per participant, representing a total of 190 measures. Thus, the total number of data used for the GEE model is quite large considering the difficulties linked with assessment of catecholamines. There were no error messages nor sign of collinearity when running the models. The level of dopamine at each day is taken in the analysis, as well as variations of correlation between variables at each measurement time.

*Dopamine* Dopamine levels were significantly lower in 24 hS at all measuring times (D1-Day, D1-Night, D3-Day and D3-night) in comparison with 14 hS and the control day.

*Norepinephrine* Norepinephrine levels at D1-Night in 24 hS and 14 hS were higher than on the control day. No differences in norepinephrine levels were reported at D1-Day and D3 between shifts and control day.

*Epinephrine* Epinephrine levels were higher at D1-Day in 24 hS and 14 hS compared with the control day. Neither *HVA* nor *VMA* differed over time.

### Stress, fatigue and sleep

Significantly higher mean scores of perceived stress, mental fatigue, and physical fatigue were observed following both the 14 hS and the 24 hS when compared to the control day. Participants reported a shorter duration of sleep during shifts (24 hS or 14 hS) compared to other days such as the day before, the day following the shift, the second day after the shift, and the control day. Moreover, the sleep duration was found to be even lower during the 24 hS compared to the 14 hS. Quality of sleep was significantly lower during shifts compared to any other day, with the lowest quality observed during the 24 hS compared to the 14 hS.

### Multivariable analysis

The consistency of our results was verified by testing each explaining variable independently, then they were also added one by one. Sensitivity analyses showed similar results. All multivariate analysis is presented in the Table 1. Regarding dopamine, a 24 hS significantly decreased levels by 44 nmol on the day 1 (*p* = 0.005), by 51 nmol on the day 3 (*p* = 0.003), by 51 nmol/L for each kg/m^2^ of BMI (*p* = 0.003), and by 9.3 nmol for every hour of napping of dopamine levels (*p* = 0.02). In contrast, males have a 71.5 nmol higher level of dopamine (*p* = 0.03); and it increased by 4.9 nmol for every nmol of HVA (*p* = 0.008). Regarding norepinephrine, 14 hS and 24 hS respectively increased the concentrations by 11 nmol (*p* = 0.03) and 12 nmol (*p* = 0.04) during the day 1. Being male was associated with a 12 nmol increase (*p* = 0.04). In contrast, for every unit increase in BMI, there was a 2.3 nmol decrease in norepinephrine (*p* = 0.01). Epinephrine levels were not influenced by the duration of the shift. For every unit increase in mental fatigue, there was a 0.1 nmol increase in epinephrine levels (*p* = 0.04); and a 0.2 nmol for every life-and-death emergency (*p* = 0.04). However, the epinephrine levels decreased by 0.1 nmol for every unit in physical fatigue (*p* = 0.005) and within the number of entries (*p* < 0.001). Similarly, for each hour of sleep, there was a 0.3 nmol decrease in epinephrine levels (*p* = 0.013). For every year in age of physicians, HVA levels increase by 0.1 nmol (*p* = 0.05); and being male was associated with a 1.1 nmol increase in HVA levels (*p* = 0.009). Similarly, for every admission, there was a 0.02 nmol increase in HVA levels (*p* = 0.03); and for every nmol of dopamine, there was a 0.01 nmol increase in HVA levels (*p* < 0.001). In contrast, every life-and-death emergency decreased HVA levels by 0.1 nmol (*p* = 0.04). Lastly, for every life-and-death emergency, there was a 0.3 nmol increase of VMA levels (*p* = 0.01); and for every admission, there was a 0.07 nmol increase of VMA levels (*p* = 0.04).

**Table 1:** Catecholamine levels: multivariable generalized estimating equations regressions.

Covariates	Dopamine			Norepinephrine			Epinephrine			HVA			VMA		
	Coeff (95% CI)		*p*	Coeff (95% CI)		*p*	Coeff (95% CI)		*p*	Coeff (95% CI)		*p*	Coeff (95% CI)		*p*
Age	− 1.9	(− 6.0, 2.2)	0.4	0.5	(− 0.2, 1.2)	0.2	0.1	(− 0.1, 0.2)	0.3	**0.1**	**(0.00, 0.1)**	**0.05**	0.01	(− 0.1, 0.2)	0.8
Male gender	**71.5**	**(8.5, 134)**	**0.030**	**12**	**(0.8, 23.3)**	**0.04**	− 1.9	(− 3.9, 0.03)	0.054	**1.1**	**(0.3, 1.9)**	**0.009**	− 1.2	(− 3.3, 1.0)	0.3
Body mass index	− **51**	**(**− **25,** − **5.1)**	**0.003**	− **2.3**	**(**− **4.2,** − **0.5)**	**0.01**	− 0.3	(− 0.6, 0.01)	0.06	− 0.1	(− 0.2, 0.1)	0.5	0.2	(− 0.2, 0.5)	0.4
Shifts, control day as REF															
14-h shift day 1	− 0.6	(− 21.5, 20.2)	0.9	**11**	**(1.0, 20.8)**	**0.03**	2.1	(− 0.9, 5.1)	0.2	− 0.2	(− 1.6, 1.2)	0.8	0.2	(− 1.6, 2.0)	0.8
14-h shift day 3	− 14	(− 40, 11.5)	0.3	1.3	(− 9.2, 11.8)	0.8	2.1	(− 0.4, 4.6)	0.1	− 0.3	(− 1.4, 0.8)	0.6	− 0.1	(− 1.9, 1.8)	0.9
24-h shift day 1	− **44**	**(**− **75,** − **13.1)**	**0.005**	**12**	**(0.6,** − **23.7)**	**0.04**	1.4	(− 1.5, 4.3)	0.3	− 0.2	(− 1.5, 1.1)	0.8	1.0	(− 1.1, 3.2)	0.3
24-h shift day 3	− **51**	**(**− **85,** − **18)**	**0.003**	− 0.6	(− 13, 11.4)	0.9	2.1	(− 0.7, 4.9)	0.1	− 0.2	(− 1.4, 1.1)	0.8	0.6	(− 1.6, 2.7)	0.6
Mental fatigue	0.8	(− 0.0, 1.5)	0.053	0.1	(− 0.2, 0.5)	0.5	**0.1**	**(0.01, 0.2)**	**0.04**	− 0.03	(− 0.1, 0.01)	0.1	0.01	(− 0.1, 0.1)	0.7
Physical fatigue	− 0.6	(− 1.4, 0.2)	0.1	0.1	(− 0.2, 0.4)	0.6	− **0.1**	**(**− **0.2,** − **0.03)**	**0.005**	0.02	(− 0.01, 0.1)	0.2	0.01	(− 0.05, 0.1)	0.7
Stress	− 0.2	(− 0.9, 0.6)	0.6	− 0.3	(− 0.6, 0.1)	0.1	0.0	(− 0.1, 0.1)	0.9	0.01	(− 0.03, 0.05)	0.7	− 0.03	(− 0.1, 0.03)	0.3
Hours of sleep	− **9.3**	**(**− **16.9,** − **1.8)**	**0.02**	− 0.7	(− 2.0, 0.7)	0.3	− **0.3**	**(**− **0.5,** − **0.1)**	**0.013**	− 0.02	(− 0.1, 0.1)	0.7	0.2	(− 0.1, 0.4)	0.2
Number of entries	− 0.9	(− 1.9, 0.2)	0.1	0.1	(− 0.1, 0.3)	0.3	− **0.1**	**(**− **0.1,** − **0.04)**	** < 0.001**	**0.02**	**(0.00, 0.03)**	**0.03**	− 0.02	(− 0.6, 0.02)	0.3
Life-and-death emergencies	− 3.5	(− 10, 3.1)	0.3	− 0.9	(− 2.1, 0.2)	0.1	**0.2**	**(0.01, 0.4)**	**0.04**	− **0.1**	**(**− **0.2,** − **0.0)**	**0.04**	**0.3**	**(0.05, 0.5)**	**0.017**
Number of admissions	− 0.6	(− 2.8, 1.6)	0.6	− 0.3	(− 0.7, 0.1)	0.1	0.05	(− 0.0, 0.1)	0.2	0.02	(− 0.01, 0.1)	0.2	**0.07**	**(0.0, 0.15)**	**0.04**
Number of beds available	− 0.0	(− 1.3, 1.3)	1.0	− 0.1	(− 0.4, 0.1)	0.2	0.02	(− 0.02, 0.05)	0.4	− 0.01	(− 0.03, 0.01)	0.2	− 0.00	(− 0.1, 0.04)	0.8
Dopamine				0.02	(− 0.0, 0.1)	0.6	− 0.01	(− 0.02, 0.00)	0.1	**0.01**	**(0.01, 0.02)**	** < 0.001**	0.01	(− 0.00, 0.02)	0.2
Norepinephrine	− 0.4	(− 0.9, 0.1)	0.1				0.02	(− 0.03, 0.1)	0.5	− 0.02	(− 0.04, 0.00)	0.1	0.03	(− 0.01, 0.07)	0.1
Epinephrine	0.8	(− 0.9, 2.5)	0.4	0.3	(− 0.5, 1.1)	0.5				0.02	(− 0.1, 0.1)	0.7	− 0.1	(− 0.2, 0.04)	0.1
HVA	**4.9**	**(1.3, 8.5)**	**0.008**	− 0.4	(− 2.2, 1.4)	0.6	0.1	(− 0.4, 0.6)	0.7				0.06	(− 0.3, 0.4)	0.7
VMA	2.4	(− 0.3, 5.1)	0.1	1.1	(0.1, 2.2)	0.1	− 0.1	(− 0.3, 0.2)	0.7	0.08	(− 0.03, 0.2)	0.2			

Significant values are in [bold].

## Discussion

The main findings of this study were that catecholamine levels were relevant biomarkers of stress during night shifts. Dopamine was the strongest biomarker with a prolonged attenuation during and following a 24 hS. This finding adds novel consequences of shift work that represents variety of impacts on the worker’s physical and psychological health, covering sleep disturbance, cardiovascular diseases, metabolic system perturbations, reproductive function decrease, oncological problems, or anxious and depressive symptoms^18^.

### Norepinephrine and epinephrine during shifts

Norepinephrine and epinephrine levels increased during shifts confirming previous findings of being associated with stress responses, to cope environmental demands^10^. Moreover, patients with depression have a higher catecholamine level^19^, and anxiety is associated with alteration of autonomic nervous system^20^, which may increase cardiovascular risk^21^. Shift work is associated with a high level of negative mood^22^. Therefore, catecholamine expressions of EPs could reflect these negative manifestations, in association with an excessive and stressful physical demand.

### Comparison between shifts

Norepinephrine and epinephrine levels at work were previously compared between day and night^12^, but no study to date have compared two types of night work. In our study, norepinephrine and epinephrine levels did not differ between 24 and 14 hS. However, dopamine levels were a strong biomarker of the 24 hS. The decrease of dopamine levels in 24 hS could be related to its role of precursor. Dopamine plays an important role in memory, cognition, motivation, or sleep^23^. Ours results suggest that 24 hS may be more detrimental to physical fatigue and attention than 14 hS. This should be considered in developing shift work policies to prevent future health complications in EPs.

### Catecholamine following shifts

We demonstrated that dopamine levels remained lower on the third day after the 24 hS than both 14 hS and control day. Despite previous findings reporting sustained biological modifications at least 3 days after a 24 hS^16,17^, we demonstrated that dopamine was also involved in a sustained and prolonged response to stress, and could differentiate between two types of night shifts. Interestingly, physical and mental fatigue remained higher after 24 hS than on the control day. As dopamine has a short half-life, a low level during the third day after the shift may mean that changes were still operant. Although not strong, literature suggests that at least 48 h were needed to recover from night work^24^. Furthermore, we propose that studies investigating biomarkers of stress should include a follow-up period of several days after a stress related event in the workplace.

### A disturbance in the circadian rhythm of catecholamine

Different hormones are involved in circadian rhythm such as norepinephrine, dopamine and melatonin^25^. The pineal gland regulates our circadian rhythm by releasing melatonin at night. The release of dopamine and norepinephrine influence the release of melatonin, leading to lower production of melatonin toward the end of the night^26^. In our study, dopamine levels remained low during and following 24 hS, suggesting a dysregulation with psychological impact such as chronic fatigue, sadness, and depression. Moreover, epinephrine was increased during the day of both shifts, whereas norepinephrine was increased during both nights of the shifts.

### Catecholamine, workload, and life-and-death emergencies

Despite some evidence of links between the number of admissions and epinephrine as well as HVA, the primary indicator of stress was the experience of life-and-death emergencies which supports our previous findings^16,17^. Life-and-death emergencies were also linked with VMA, and demonstrated a stronger link with catecholamine than the number of admissions. Although speculative, our findings could potentially establish a connection to the elevated morbidity observed in patients following instances where cardiac surgeons have experienced a patient’s death during emergency surgery^27^.

### Catecholamine, fatigue, sleep, sex, and obesity

Epinephrine appeared to be the most relevant fatigue-related catecholamine, which is consistent with literature^28^. Despite low levels of dopamine during a 24 hS, we confirmed previous findings demonstrating that dopamine increased after sleep deprivation^29^ and responses may reflect its role as a precursor within the catecholamine pathway. The catecholamine response to shifts was mostly increased in males, as previously shown^30^. This could suggest the high prevalence of women working in the EDs. In our study, BMI was inversely associated with dopamine and norepinephrine levels, which is consistent with the literature^31^. Despite the absence of obesity among EPs in our study, EPs with high BMI may have delayed reactions to emergency events. It is well accepted that catecholamine metabolites vary from birth to adulthood, and we also reported that HVA seemed to be linked with age. A larger sample size could provide more detailed sex specificity of stress responses in EPs, considering BMI and age.

### Limitations

Our study has some limitations. First, external validity may not extend to EDs that have less intensive workloads—mainly represented by the number of daily ED visits and the severity of the patients, two parameters not assessed in our study—and may also lack some external validity due to the single site design. However, we were able to find significant and prolonged impact of shift among EPs. Secondly, the sample size may seem small for the number of variables tested in the GEE model, but our study incorporated several methodological strengths. We computed our analysis using a stepwise approach, by adding variables one by one to search for putative bias. Moreover, even if we included only 19 emergency physicians, we measured urinary levels of catecholamine twice each 24-h of collection, over 5 days i.e. GEE models have in fact 190 measures for each catecholamine. We also designed a randomized order of shifts, a run-in design, participants acting as their own controls, a sufficient statistical power to support the primary hypothesis, identification of factors influencing catecholamine levels, objective and subjective assessment of workload, and the innovative aspect of being the one of the first studies to compare two types of shift while also monitoring participants during a recovery period. The duties and shifts of EPs may vary across the world, yet there is limited research on systemic responses to life-and-death emergencies. Our study results offer valuable insights and opportunities to generate hypotheses for future research in this area.

## Conclusion

Urinary catecholamine is a convenient, non-invasive, and strong biomarker of stress, sleep deprivation and mental fatigue both acutely and over time. Dopamine was lowest during 24 hS. Furthermore, we observed that 24 hS contributed to a prolonged response in dopamine, leading to decreased catecholamine levels at least 3 days after the shift. Norepinephrine and epinephrine were highest during the 14 hS and 24 hS, only on the day of the shift. Epinephrine appears to be associated to mental fatigue. Considering the association between elevated catecholamine levels, cardiovascular disease, and negative psychological consequences, we recommend that EPs discontinue the practice of 24 hS.

## Data Availability

All relevant data are within the paper.
